# A Case of Secondary Sclerosing Cholangitis in the setting of Non-Hodgkin’s Lymphoma

**DOI:** 10.7759/cureus.4707

**Published:** 2019-05-21

**Authors:** Hira Shaikh, Shifa Umar, Moaz Sial, Antonios Christou, Abhijit Kulkarni

**Affiliations:** 1 Internal Medicine, Allegheny Health Network, Pittsburgh, USA; 2 Gastroenterology, Hepatology and Nutrition, Allegheny Health Network, Pittsburgh, USA; 3 Hematology and Oncology, Allegheny Health Network, Pittsburgh, USA

**Keywords:** secondary sclerosing cholangitis, non-hodgkin’s lymphoma, anaplastic large cell lymphoma, biliary tract obstruction

## Abstract

Sclerosing cholangitis represents a spectrum of cholestatic liver disease characterized by inflammation, fibrosis, and stricture of the bile ducts. A 67-year-old Caucasian female with a history of breast cancer in remission, presented with jaundice and an exophytic mass at the base of the tongue. Laboratory data revealed cholestasis with alkaline phosphatase 953 U/L, total bilirubin 7.7 mg/dL, direct bilirubin 6.4 mg/dL, and gamma-glutamyltransferase 3369 U/L. Computed tomography (CT) scan showed widespread lymphadenopathy in the chest, abdomen, and pelvis concerning for lymphoma, acute pancreatitis and biliary dilation with hyperenhancement of the common bile duct wall. Diffuse intrahepatic biliary ductal dilatation and narrowing with multifocal stenosis of the proximal and distal aspects of the common bile duct was seen on magnetic resonance cholangiopancreatography (MRCP). Findings were consistent with sclerosing cholangitis. Pathology of the oral lesion revealed activin receptor-like kinase 1 (ALK1) positive anaplastic large cell lymphoma. Chemotherapy was initiated with cyclophosphamide, doxorubicin, adriamycin, vincristine, etoposide, and prednisone (CHOEP-14) regimen, which resulted in significant clinical improvement along with a remarkable decrease in the liver function tests. Non-Hodgkin’s lymphoma (NHL) has only rarely been reported in the literature as a cause of secondary sclerosing cholangitis, i.e., only 0.2% to 2.0% of patients with NHL present with biliary tract obstruction. It is essential for gastroenterologists, oncologists, and radiologists to recognize sclerosing cholangitis occurring secondary to a systemic disease because early initiation of treatment can improve clinical outcome, as manifested by our case.

## Introduction

Sclerosing cholangitis represents a spectrum of cholestatic liver disease characterized by inflammation, fibrosis, and stricture of the bile ducts. Primary sclerosing cholangitis (PSC) is the most common type. Diagnosis requires the exclusion of secondary causes of sclerosing cholangitis and an understanding of conditions that may potentially mimic its classic cholangiographic features [1]. These entities should be on the differential and not be missed, because they may be reversible and respond favorably to therapy. Lymphoma is a rare cause of extrahepatic obstruction. Previous studies have reported 1.3% of patients with lymphoma can develop extrahepatic biliary obstruction with the most common cause being compression of the extrahepatic biliary tract due to enlarged lymph nodes [2-4]. Other etiologies include Non-Hodgkin’s lymphoma (NHL) arising from the extrahepatic bile duct, primary involvement of the common bile duct (CBD) by Hodgkin’s lymphoma (HL), primary lymphoma arising from the porta hepatis or the ampulla of Vater, primary lymphoma of the pancreas involving the CBD by local infiltration and gastrointestinal lymphoma obstructing the bile duct at the ampulla of Vater. Herein, we present a case of secondary sclerosing cholangitis in the setting of anaplastic large cell lymphoma. 

## Case presentation

A 67-year-old Caucasian female with a past history of breast cancer status post lumpectomy, radiation, and hormonal therapy, presented with jaundice and an exophytic mass at the base of the tongue. Laboratory data revealed alkaline phosphatase 953 U/L, total bilirubin 7.7 mg/dL, direct bilirubin 6.4 mg/dL, gamma-glutamyltransferase 3369 U/L, aspartate aminotransferase 195 U/L, alanine aminotransferase 149 U/L, albumin 3.1 g/dL, and protein 6.9 g/dL (Abstract: Umar S, Sial M, Christou A, Kulkarni A. A Case of Secondary Sclerosing Cholangitis in the Setting of Non-Hodgkin’s Lymphoma. ACG; 2016). A computed tomography (CT) scan of the chest, abdomen, and pelvis showed widespread lymphadenopathy in the chest, abdomen, and pelvis concerning for lymphoma. It also showed acute pancreatitis and biliary dilation with the hyperenhancement of the common bile duct wall. 

**Figure 1:**
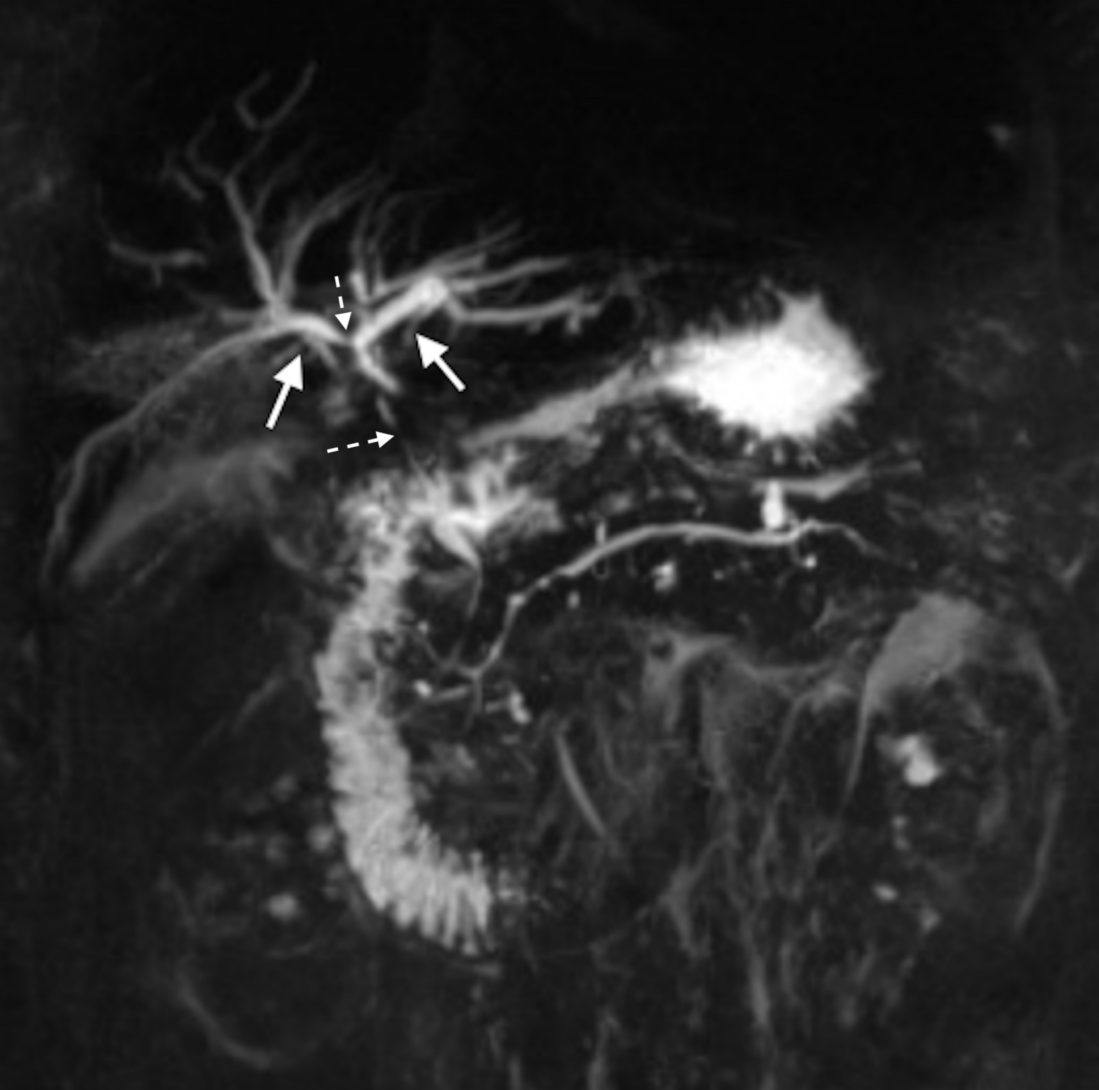
Diffuse biliary dilatation (solid arrows) with sclerosis (broken arrows) seen on MRCP MRCP, magnetic resonance cholangiopancreatography

Magnetic resonance cholangiopancreatography (MRCP) was performed, which showed diffuse intrahepatic biliary ductal dilatation with non-visualization of the common hepatic duct bifurcation. Diffuse narrowing with multifocal stenosis of the proximal and distal aspects of the common bile duct was also seen (Figure 1). 

Findings were consistent with sclerosing cholangitis. Pathology of the oral lesion revealed activin receptor-like kinase 1 (ALK1)-positive anaplastic large cell lymphoma, a type of non-Hodgkin’s lymphoma. Chemotherapy was initiated with cyclophosphamide, doxorubicin, adriamycin, vincristine, etoposide, and prednisone (CHOEP-14) regimen, which resulted in significant improvement in liver function tests. The patient continued to improve clinically and was discharged with close outpatient follow-up. 

## Discussion

Sclerosing cholangitis, a chronic cholestatic liver disease, occurs due to immune-mediated destruction and fibro-obliteration resulting in strictures of extrahepatic and intrahepatic bile ducts, and eventually biliary cirrhosis [5]. Primary sclerosing cholangitis (PSC) is of unknown etiology, and to date, besides symptomatic management, no specific treatment has been identified [6]. In contrast, secondary sclerosing cholangitis (SSC) has been commonly reported in the literature to occur in association with various conditions, such as biliary tract stone disease, autoimmune pancreatitis, abdominal trauma, eosinophilic cholangitis, and recurrent pyogenic cholangitis among many others [1]. While NHL has frequently been associated with autoimmune disease (7.6%), it has infrequently been found to be the cause of malignant biliary obstruction (1-2%) [6-7]. Cholangiographic evidence is the standard and liver biopsy is usually not required [5].

Including the various mechanisms through which NHL can cause cholestasis, extrahepatic obstruction secondary to lymphadenopathy is the most commonly reported one [3-4]. Others include direct intrahepatic invasion by lymphoma, tumor-related hemolysis, idiopathic cholestasis, and biliary obstruction [8].

Because we were not able to find any cause beside NHL, and also due to a good response to the NHL treatment, we speculated that sclerosing cholangitis in our case was associated with the lymphoma. There has been some evidence that dysfunctional immune system, similar to the case of autoimmune diseases, may give rise to a lymphoma [6]. Because both NHL and sclerosing cholangitis were diagnosed at the same time in our case, similar to what other studies have demonstrated, there is no way of knowing which played a role in the pathogenesis of the other.

Although the bile duct may be compressed by the lymphomatous mass anywhere along its path, the most common sites of obstruction are the hilum (by porta hepatis lymph nodes) and the distal common bile duct (by peripancreatic nodes) [9]. At these locations, the bile duct is relatively fixed and is hard to be displaced. This adds to the challenge, because these areas are not easily accessible for biopsy, thus making the diagnosis difficult. However, recent studies have advocated more promise in the histopathological diagnosis owing to the improved imaging techniques and the safety of image-guided needle biopsy [10].

Studies have demonstrated that in such cases, chemotherapy is the mainstay of treatment and can achieve complete remission in almost half of the patients involved in the study [3]. Meanwhile, there have been cases in which endoscopic or percutaneous stent placement was used for biliary drainage resulting in resolution of hyperbilirubinemia [3-4].

There have been reports of recurrent cholangitis after achieving a complete remission with chemotherapy. After a recurrence of disease was excluded through imaging, the cause proved to be a benign biliary stricture at the site of prior lymphoma, treated with a biliary stent. These cases emphasize the need for extended follow-up after successful chemotherapy for possible benign biliary stricture formation [3-4].

NHL as a secondary cause of sclerosing cholangitis has rarely been reported in the literature, with the incidence being low. The diagnosis of extra-hepatic biliary obstruction related to lymphoma is challenging because it usually presents at the time of diagnosis, is difficult to differentiate on cholangiography from benign stricture, primary sclerosing cholangitis, cholangiocarcinoma, pancreatic carcinoma and usually occurs at hilum or peripancreatic area, which are anatomically difficult to biopsy [2].

Biliary obstruction and patients who develop jaundice as a late symptom in lymphoma usually appears in the advanced stage of the disease [3-4]. While our patient had a good response after the chemotherapy, biliary obstruction is a sign of poor prognosis in lymphoma [4,9]. This suggests that these patients are usually in an advanced stage of the disease and should be diagnosed early. The course of our patient concurs a novel manifestation that, if diagnosed early and managed aggressively, remarkable recovery and a good prognosis is achievable.

## Conclusions

Diagnosing secondary cholangitis and differentiating it from a local versus systemic process can be challenging. This report emphasizes the importance of consideration of lymphoma, particularly non-Hodgkin’s lymphoma, in the diagnosis of biliary obstruction resembling sclerosing cholangitis due to their frequent co-occurrence. Also, the co-existence of both conditions comes with high but preventable mortality. If delivered in time, chemotherapy has shown promising results with favorable prognosis. Therefore, we strongly recommend that the possibility of non-Hodgkin’s lymphoma should be considered when working up patients for secondary sclerosing cholangitis.
